# Case Report: IgG4-related lung disease

**DOI:** 10.3389/fimmu.2026.1897950

**Published:** 2026-09-28

**Authors:** Yehui Li, Yuanbin Luo, Liwen Zhou, Xianmei Zhou

**Affiliations:** 1 Department of Respiratory and Critical Care Medicine, Affiliated Hospital of Nanjing University of Chinese Medicine, Nanjing, Jiangsu, China; 2 Department of Pathology, Gansu Provincial Hospital of Traditional Chinese Medicine, Lanzhou, Gansu, China; 3 Department of Pulmonary Disease, Gansu Provincial Hospital of Traditional Chinese Medicine, Lanzhou, Gansu, China

**Keywords:** case report, IgG4-related lung disease, imaging findings, multiple systems, pathological features

## Abstract

Immunoglobulin G4-related disease (IgG4-RD) is a systemic immune-mediated fibroinflammatory condition, distinct from classical autoimmune disorders, that can affect multiple organs. Pulmonary involvement often presents with non-specific clinical and radiographic features, contributing to high rates of misdiagnosis and underdiagnosis. Here, we report a rare case of IgG4-related lung disease (IgG4-RLD) to enhance clinical awareness. A 71-year-old female presented with cough. Chest computed tomography (CT) revealed an irregular soft tissue mass in the right middle lobe. All autoantibodies tested were negative, and the serum IgG4 level was within the normal range. The patient showed no response to anti-infective and antitussive therapy, with follow-up CT confirming lesion enlargement. A CT-guided percutaneous lung biopsy was subsequently performed. Histopathology demonstrated infiltration of numerous lymphocytes and plasma cells, along with scattered neutrophils. Immunohistochemistry showed a ratio of IgG4-positive to IgG-positive plasma cells of approximately 42%, and demonstrated approximately 30 IgG4-positive plasma cells per high-power field (HPF). The diagnosis of IgG4-RLD was established based on the 2019 ACR/EULAR International Classification Criteria for IgG4-RD. The patient was subsequently treated with oral prednisone, which led to complete resolution of the cough and near-complete regression of the pulmonary lesion.

## 1 Introduction

Immunoglobulin G4-related disease (IgG4-RD) is an immune-mediated chronic fibro-inflammatory disease that has only been gradually recognized in recent years. Its histological features include dense lymphoplasmacytic infiltration, storiform fibrosis, and obliterative phlebitis (1), with elevated serum IgG4 levels found in only about 50% of patients (2). Almost any organ or tissue can be affected, presenting as single-organ or multi-organ involvement, including the pancreas, hepatobiliary system, salivary glands, lacrimal glands, kidneys, and retroperitoneum (3). Research by Wallace ZS et al. indicates that the distribution of organs affected by IgG4-RD differs between Asian and non-Asian cases; individuals of Asian descent are particularly prone to IgG4-RD complications in the head and neck. In contrast, non-Asian patients are more likely than Asian patients to have pancreato-hepatobiliary disease and/or retroperitoneal and aortic disease (3). Notably, the first discovery of IgG4-RD-related pancreato-hepatobiliary disease occurred in Japan (4). The literature on IgG4-related lung disease (IgG4-RLD) is limited, consisting only of case reports and small series. In 2004, Duvic et al. first reported IgG4-related lung disease (5), describing a case of multifocal idiopathic fibro-sclerosis with retroperitoneal fibrosis, pancreatic fibrosis, and obliterative bronchiolitis combined with organizing pneumonia. IgG4-RLD mainly consists of four clinical lung syndromes: inflammatory pseudotumor of the lung, central airway disease, interstitial pneumonia, and pleuritis. Characteristic imaging features of the lungs include nodules, ground-glass opacities, alveolar and interstitial lesions, and bronchovascular bundle thickening (1). Symptoms are wide-ranging and nonspecific, including cough, dyspnea, fever, and chest pain. Therefore, IgG4-RD affecting the lungs needs to be differentiated from various other diseases, such as other immune-mediated diseases (such as vasculitis, sarcoidosis), infections, and tumors (such as lymphoma, lung cancer).

The current standard of care for IgG4-RD is glucocorticoid therapy, which may be administered with or without disease-modifying antirheumatic drugs (6). It has been well established that pulmonary involvement in IgG4-RD is highly responsive to prednisone, with notable amelioration of both symptoms and radiographic abnormalities (7). Continuous use of low-dose glucocorticoids can prevent long-term relapse (8, 9). Due to the overall rarity of IgG4-RD, non-specific clinical symptoms, and difficulty in obtaining tissue samples, definitive diagnosis remains challenging. The limited literature on IgG4-RLD often leads to missed or incorrect diagnoses, which may result in delayed treatment (10, 11), ultimately causing long-term complications or unnecessary examinations, increasing patients’ symptom burden and morbidity. Therefore, correct and timely diagnosis of this type of disease is crucial for patient treatment and prognosis. Herein, we report an atypical case of IgG4-RLD from our hospital to enhance clinicians’ awareness of this condition.

## 2 Case presentation

This case report follows the CARE (CAse REport) guidelines. A 71-year-old female presented with intermittent cough for 20 days. The patient reported that 20 days before the initial clinical presentation, she developed a cough with no identifiable precipitating factor, producing a small volume of thin, white, frothy sputum, accompanied by dryness and soreness of the throat. She also experienced chest tightness, exertional dyspnea, fatigue, nausea, oral sticky sensation and diaphoresis, with no associated chills, fever, nasal congestion or rhinorrhea. Her medical history included hypertension and type 2 diabetes. She denied any history of infectious diseases, food or drug allergies, smoking, or dust exposure. On admission, her vital signs were: temperature 36.6 °C, heart rate 105 bpm, respiratory rate 20 breaths/min, blood pressure 174/96 mmHg, and peripheral oxygen saturation 90% on room air. Physical examination revealed bilateral submandibular gland enlargement. Chest percussion revealed clear sounds bilaterally; auscultation demonstrated slightly decreased breath sounds in the right lung and clear breath sounds in the left lung, with no obvious dry or moist rales.

Laboratory findings on admission revealed a white blood cell count (WBC) of 11.68 × 10^9^/L with an absolute neutrophil count of 8.32 × 10^9^/L, elevated c‑reactive protein (CRP) (35.69 mg/L), and erythrocyte sedimentation rate (ESR) (23 mm/h). Complement component 3 (C3) was elevated (1.8 g/L), while immunoglobulins (IgA, IgM, IgG) and complement component 4 (C4) were within normal limits. Tumor markers showed mildly elevated carbohydrate antigen 199 (CA199) (50.0 U/mL) and carcinoembryonic antigen (CEA) (3.55 ng/mL). A comprehensive panel of autoantibodies (including ANA, anti‑CCP, anti‑SSA/SSB, anti‑dsDNA, anti‑Sm, anti‑Ro52, and anti‑PM‑Scl, etc.), vasculitis‑related antibodies (c‑ANCA, p‑ANCA, anti‑GBM, anti‑MPO, and anti‑PR3), and infectious markers (G test, GM test, and tuberculosis antibody) were all negative. A complete summary of all laboratory findings is provided in **Table 1**.

**Table 1:** Summary of laboratory findings at initial presentation.

Parameter	Result	Reference range
Arterial blood gas analysis (room air, FiO_2_ 21%)		
PH	7.40	7.35-7.45
PO_2_ (mmHg)	67	83-108
PCO_2_ (mmHg)	34.6	35-45
cK^+^ (mmol/L)	3.69	3.40-4.50
cNa^+^ (mmol/L)	138	136-146
cCl^−^ (mmol/L)	107	98-106
cLac (mmol/L)	1.1	0.5-1.6
P/F ratio (mmHg)	319	≥300
Complete blood count		
WBC (×10^9^/L)	**11.68**	3.5-9.5
Absolute neutrophil count (×10^9^/L)	**8.32**	1.8-6.3
Other parameters	Normal	—
Inflammatory markers		
CRP (mg/L)	**35.69**	0-6
ESR (mm/h)	**23**	0-20
Tumor markers		
AFP (ng/mL)	2.15	0-7.02
CEA (ng/mL)	**3.55**	0-3.4
CA199 (U/mL)	**50.00**	0-39
CA125 (U/mL)	11.20	0-35
CA153 (U/mL)	5.44	0-25
NSE (ng/mL)	7.489	0-23
CYFRA21-1(ng/mL)	2.29	0-3.3
SCCA (ng/mL)	0.29	0.2-70
Immunological markers		
C3 (g/L)	**1.8**	0.7-1.4
C4 (g/L)	0.37	0.1-0.4
IgA (g/L)	3.48	1.0-4.2
IgM (g/L)	1.50	0.5-2.8
IgG (g/L)	12.89	8.6-17.4
Lymphocyte subsets		
Total T lymphocytes (%)	**85.82**	50.00–84.00
Helper T cells (%)	**26.77**	27.00–51.00
Cytotoxic T cells (%)	**57.29**	15.00–44.00
B lymphocytes (%)	**2.66**	5.00–18.00
Helper T cell/Cytotoxic T cell ratio	**0.47**	0.71–2.78
Infection-related tests		
G test	Negative	—
GM test	Negative	—
Autoantibody panel		
ANA, anti-Ro52, anti-CCP, anti-SSA, anti-SSB, anti-Jo-1, anti-Scl-70, anti-dsDNA, anti-nucleosome, anti-histone, anti-ribosomal P, anti-PM-Scl, anti-Sm, AMA-M2	Negative	—
Vasculitis-related antibody panel		
c-ANCA	Negative	—
p-ANCA	Negative	—
anti-GBM	Negative	—
anti-MPO	Negative	—
anti-PR3	Negative	—
Others		
Coagulation function	Normal	—
Thyroid function	Normal	—
Liver and renal function	Normal	—
Routine urine and stool tests	Normal	—

WBC, White blood cell count; CRP, C-reactive protein; ESR, Erythrocyte sedimentation rate; AFP, Alpha-fetoprotein; CEA, Carcinoembryonic antigen; CA199, Carbohydrate antigen 199; CA125, Carbohydrate antigen 125; CA153, Carbohydrate antigen 153; NSE, Neuron-specific enolase; CYFRA21-1, Cytokeratin 19 fragment; SCCA, Squamous cell carcinoma antigen; C3, Complement component 3; C4,Complement component 4; IgA, Immunoglobulin A; IgM, Immunoglobulin M; IgG, Immunoglobulin G; ANA, Antinuclear antibody; CCP, Cyclic citrullinated peptide antibody; SSA, Sjögren’s-syndrome-related antigen A antibody; SSB, Sjögren’s-syndrome-related antigen B antibody; dsDNA, Double-stranded DNA antibody; Scl-70, Topoisomerase I antibody; Jo-1, Histidyl-tRNA synthetase antibody; PM-Scl, Polymyositis/Scleroderma antibody; AMA-M2, Anti-mitochondrial antibody M2; c-ANCA, Cytoplasmic anti-neutrophil cytoplasmic antibody; p-ANCA, Perinuclear anti-neutrophil cytoplasmic antibody; GBM, Glomerular basement membrane antibody; MPO, Myeloperoxidase antibody; PR3, Proteinase 3 antibody.Bolded values indicate abnormal readings.

The enhanced chest CT shows an irregular soft tissue density mass in the lateral segment of the right middle lung lobe, surrounded by streaks, measuring approximately 29×23×32mm. On enhanced scanning, patchy unenhanced low-density areas are visible within it, with progressive enhancement of the surrounding solid components. Multiple nodules are present in both lungs, some with calcification. Streaks are seen in the lingular segment of the left upper lobe and the medial segment of the right middle lobe. Multiple enlarged lymph nodes are present in the right pulmonary hilum, and multiple lymph nodes are observed in both axillae. Aortic and coronary artery atherosclerosis is noted, along with bilateral pleural thickening. A small amount of pleural effusion is present on the right side (**Figure 1**). Pulmonary function testing revealed a mild mixed ventilatory impairment, while the diffusion capacity was normal.

**Figure 1:**
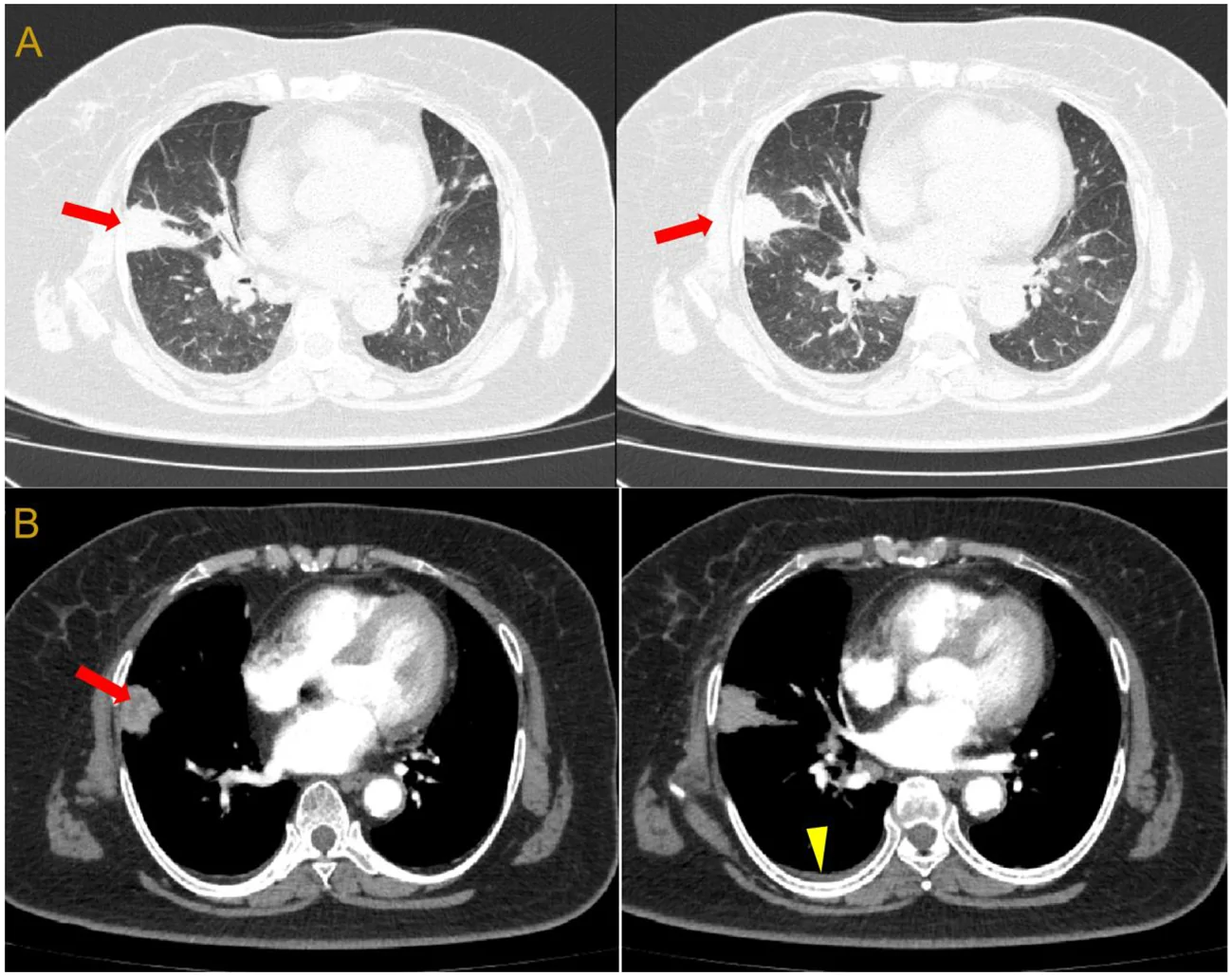
Chest CT performed on August 7, 2023. **(A)** Lung window; **(B)** Mediastinal window. The right middle lobe mass (red arrow) shows patchy non‑enhanced low‑density areas with progressive enhancement of the surrounding solid component. A small amount of pleural effusion is present in the right thoracic cavity (yellow triangle).

The patient was treated with piperacillin sodium for anti-infection therapy and symptomatic treatment for cough. However, the patient’s cough symptoms did not improve, so a electronic bronchoscopy was performed on the third day of hospitalization. Bronchoscopic findings included: congestion and edema of the mucosa in the lumens of all lobar segments, normal openings, no stenosis, no neoplasms, visible longitudinal folds, and carbon-like deposits on some bronchial walls. Full-course suctioning was performed under electronic bronchoscopy. A brush biopsy and bronchoalveolar lavage (BAL) were performed on the lateral segment of the right middle lobe (**Figure 2**). Bronchial brush cytology showed no malignant cells. Both bacterial and fungal cultures of the BAL fluid were negative, and an Xpert MTB/RIF assay also returned negative for *Mycobacterium tuberculosis* DNA. Subsequently, with the patient’s consent, a CT-guided percutaneous lung biopsy was performed. During the wait for histopathological results, the patient’s cough and exertional shortness of breath worsened. A follow-up chest CT performed on August 22, 2023 further enlargement of the right lung lesion (from approximately 29×23×32 mm to approximately 33×24×33 mm), as well as an increase in bilateral pleural effusion (**Figure 3**). The histopathological results showed: widened alveolar septa, fibroblast proliferation in some areas, infiltration of numerous lymphocytes and plasma cells, and a small number of neutrophils. Immunohistochemistry showed a ratio of IgG4-positive to IgG-positive plasma cells of approximately 42%, and demonstrated approximately 30 IgG4-positive plasma cells/HPF (**Figure 4**). These pathological features were consistent with IgG4-RLD. The serum IgG4 level of the patient was within the normal range. Color Doppler ultrasound revealed multiple enlarged lymph nodes in the bilateral submandibular glands, parotid glands, neck, axillae, and inguinal regions. The the patient was considered to have IgG4-RLD, and treatment with prednisone acetate was initiated at a single daily dose of 60 mg.

**Figure 2:**
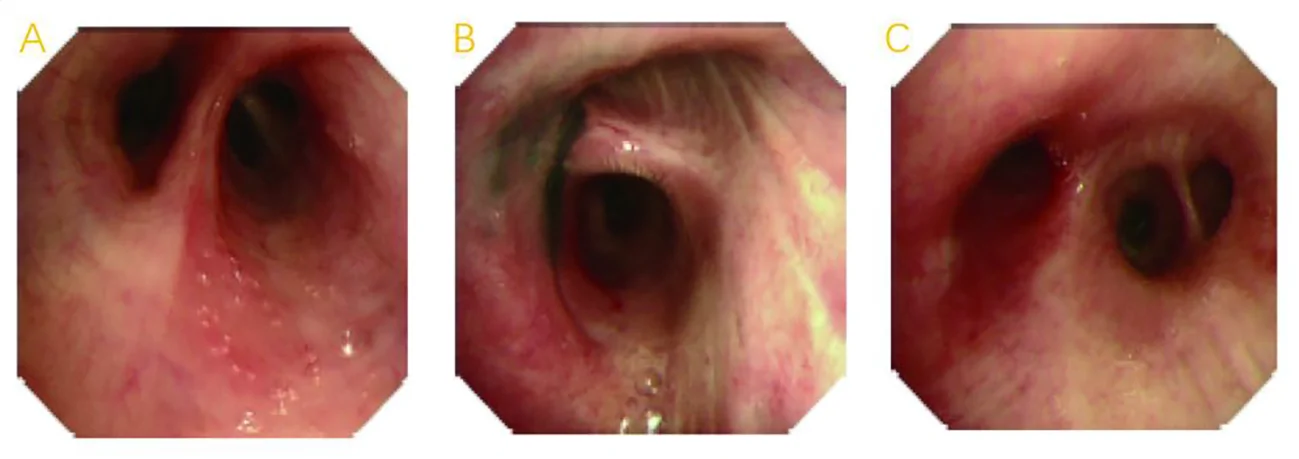
**(A)** Right middle lobe of the lung; **(B)** Right bronchus; **(C)** Right middle segment bronchus. Electronic bronchoscopy showed mucosal congestion and edema in the lumen, visible longitudinal folds, and carbon-like deposits on some parts of the bronchial wall.

**Figure 3:**
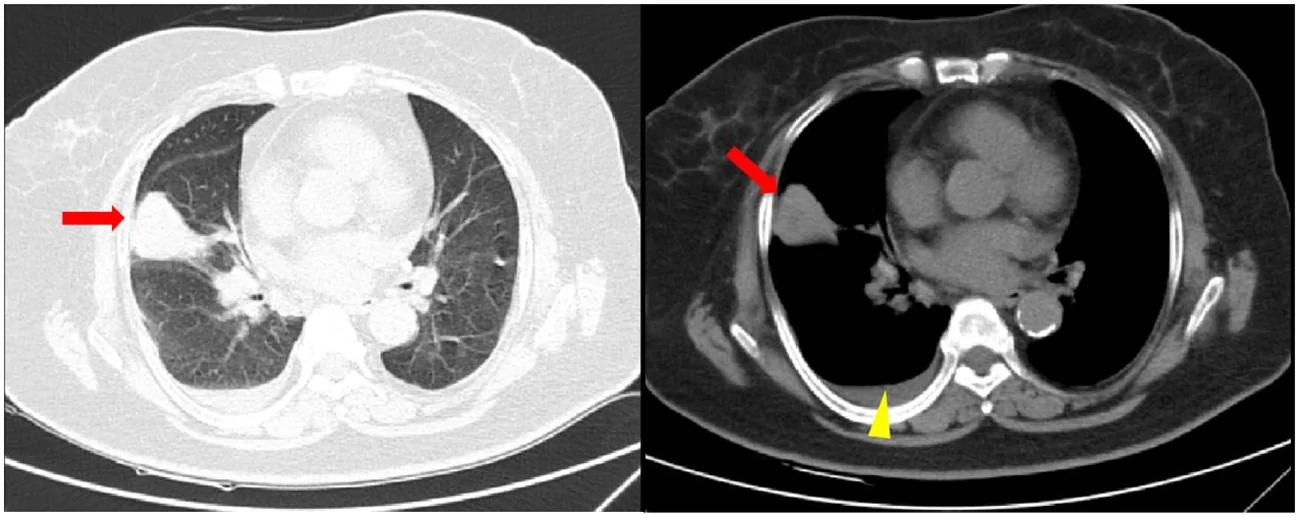
Follow-up chest CT performed on August 22, 2023. The right middle lobe mass has enlarged (red arrow), and the right pleural effusion has increased (yellow triangle).

**Figure 4:**
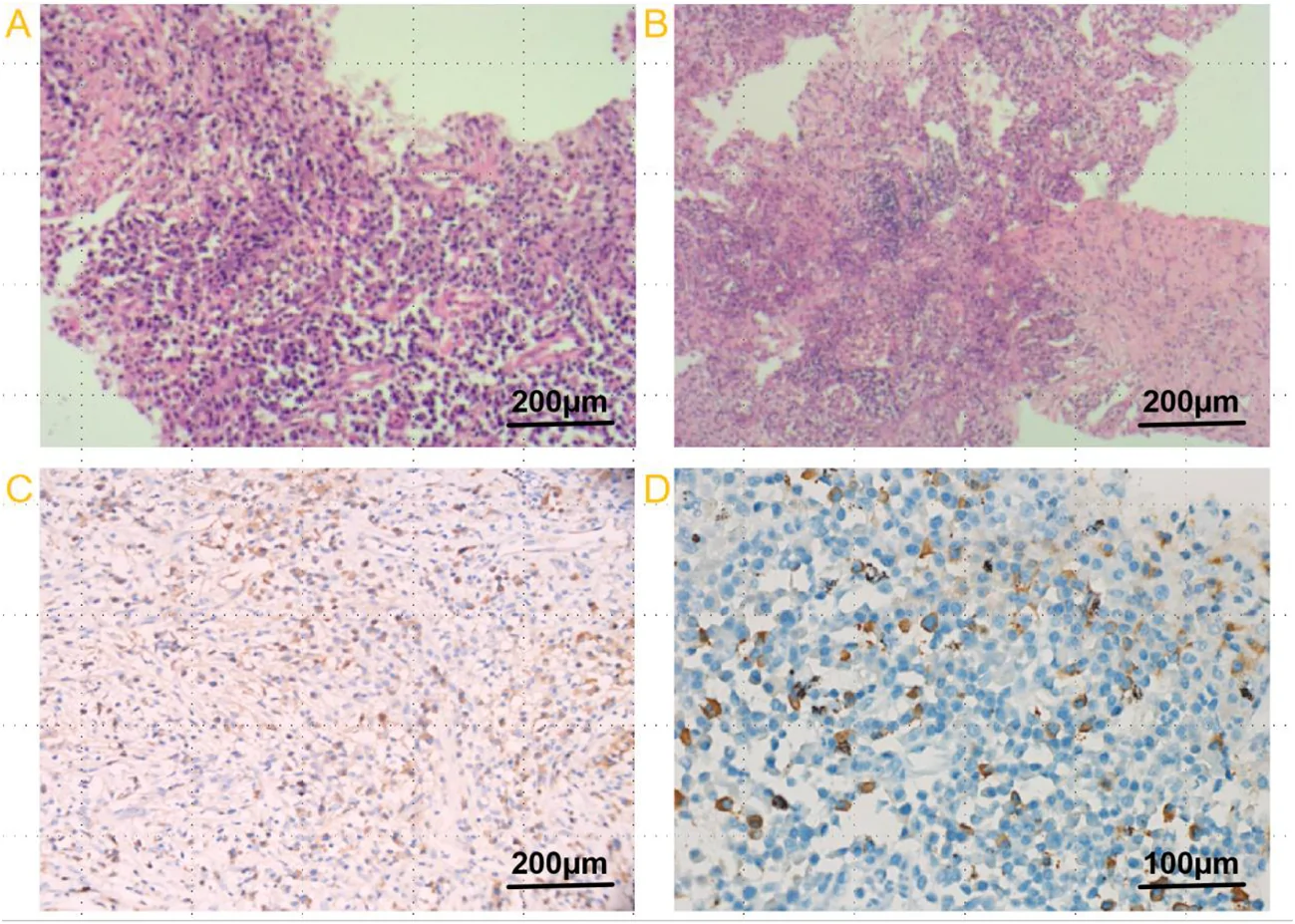
Pathological features of lung tissue biopsy. **(A–B)** HE staining revealed infiltration of lymphocytes and plasma cells in the tissue slice (scale bar = 200 μm; original magnification ×200). **(C)** Immunohistochemical staining showed IgG-positive plasma cells in the tissue slice (scale bar = 200 μm; original magnification ×200). **(D)** Immunohistochemical staining revealed IgG4-positive plasma cells in the tissue slice (scale bar = 100 μm; original magnification ×400).

After 10 days of oral prednisone treatment, follow-up chest CT showed reduction of the lesion compared with previous imaging, and the right pleural effusion had also decreased (**Figure 5**). The prednisone acetate was then tapered as follows: reduced to 50 mg/day, followed by a reduction of 10 mg every 2 weeks until reaching 20 mg/day; after maintaining 20 mg/day for 2 weeks, the dose was further reduced by 5 mg every 2 weeks until reaching a maintenance dose of 5 mg/day. A subsequent chest CT on October 9 revealed a marked decrease in the size of the lesion in the right middle and lower lobes, measuring approximately 17×10×24 mm (**Figure 6**). Approximately 3 months after the October 9 scan, a third chest CT performed at an external hospital showed that the lung lesions had been almost completely resolved (**Figure 7**). **Table 2** summarizes the diagnostic and treatment course of this case.

**Figure 5:**
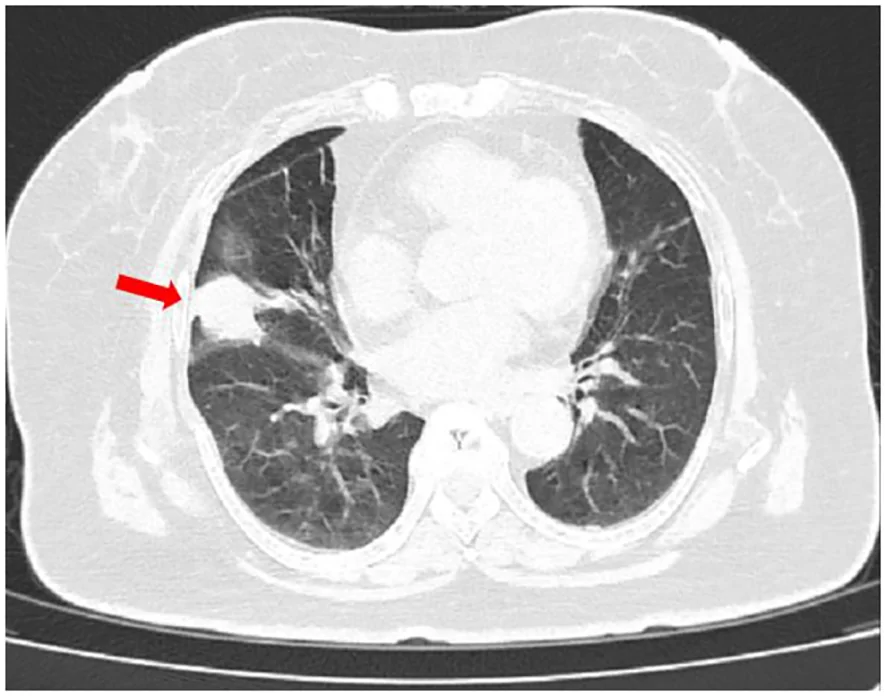
Follow-up chest CT performed on September 1, 2023. Compared with **Figure 3**, the right middle lobe mass has decreased in size (red arrow), and the right pleural effusion has reduced.

**Figure 6:**
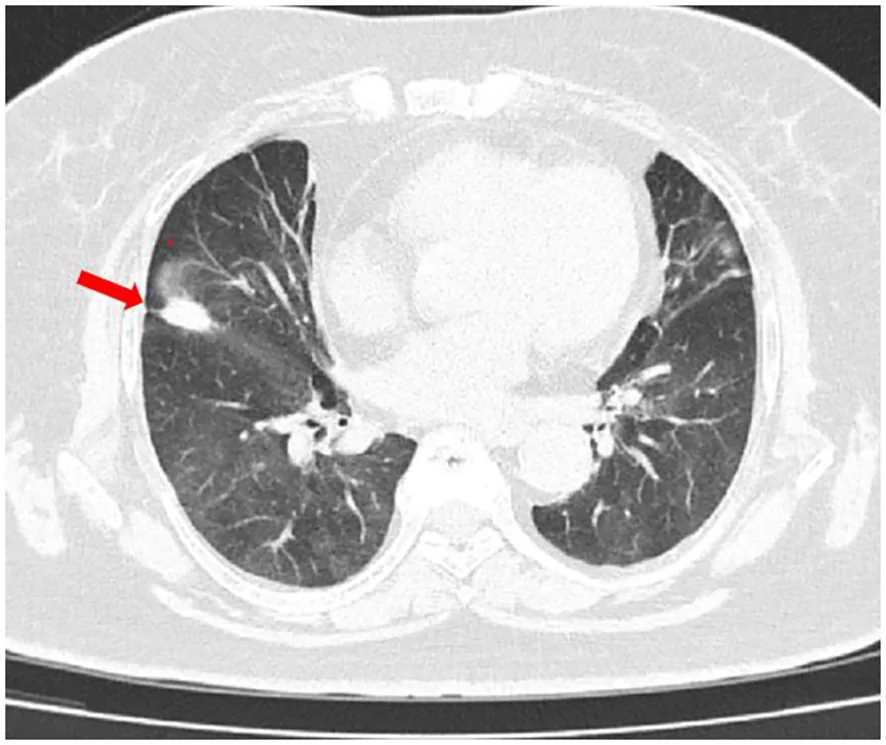
Follow-up chest CT performed on October 9, 2023.

**Figure 7:**
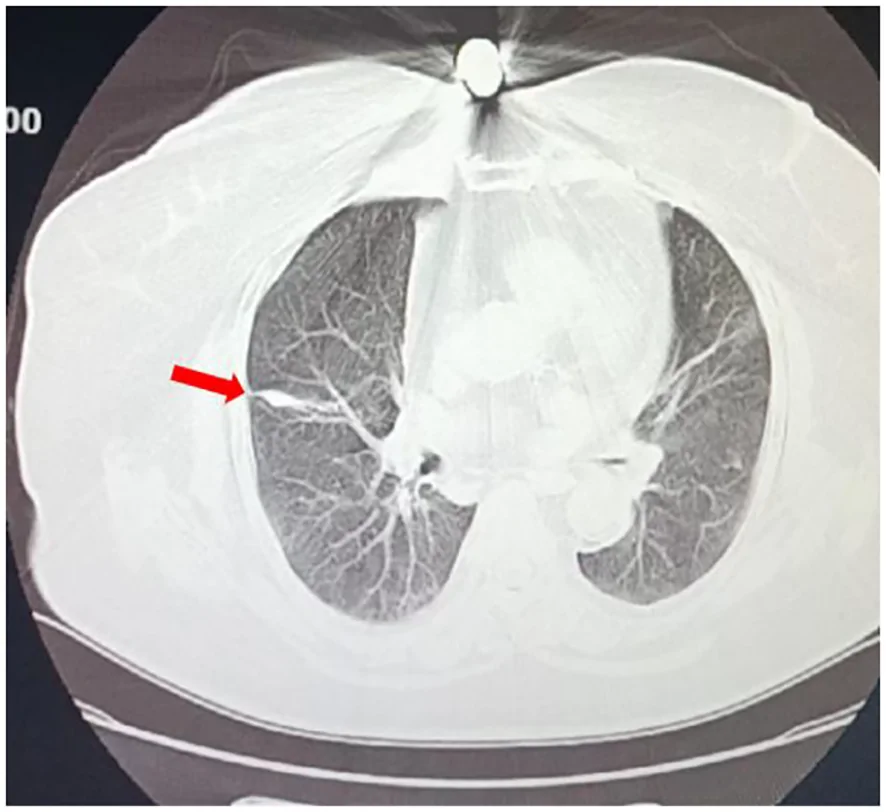
Follow-up chest CT performed at an external hospital in January 2024 (approximately 3 months after **Figure 6**).

**Table 2:** Timeline of clinical events, diagnostics, and treatment.

Date	Key clinical event	Summary of findings/outcome
2023-08-07	Admission and initial presentation	Cough, productive sputum, fatigue; bilateral submandibular gland enlargement, SpO_2_ 90%. Initial chest CT: irregular right middle lobe mass (29×23×32 mm), bilateral nodules, right hilar lymphadenopathy, small right pleural effusion.
2023-08-09	Bronchoscopy and lung biopsy	Bronchoscopy: mucosal congestion, no mass; BAL negative for infection/malignancy. CT‑guided percutaneous lung biopsy performed.
2023-08-18	Pathological diagnosis	Pathology: widened alveolar septa, lymphoplasmacytic infiltration; IgG4+/IgG+ plasma cell ratio: ~42%; IgG4+ plasma cells ~30/HPF. Serum IgG4 normal. 2019 ACR/EULAR classification >20 → IgG4‑RLD.
2023-08-22	Treatment initiation	Started oral prednisone 60 mg/day.
2023-09-01	First follow‑up: partial clinical and radiographic response	Symptoms: Markedly reduced cough. Imaging: Chest CT – right lung mass decreased (partial response).
2023-10-09	Second follow‑up: significant clinical and radiographic improvement	Symptoms: Minimal cough. Imaging: Chest CT – mass significantly reduced (17×10×24 mm) → significant improvement.
2024-01	Final follow‑up: near‑complete clinical and radiographic remission	Symptoms: Nearly asymptomatic. Imaging: Chest CT (external hospital) – pulmonary lesion nearly completely resolved → near‑complete remission.

## 3 Discussion

IgG4-RD is a chronic systemic immune-mediated fibroinflammatory condition. The prevalence of IgG4-RD is approximately 0.8 per 100,000, with a male predominance (62%–83%) (12). Although the disease was initially found to primarily affect the pancreas, the range of affected organs varies from the orbit to the retroperitoneum. Pulmonary involvement, termed IgG4-related lung disease (IgG4-RLD), was first reported in 2004 (5). The true incidence of IgG4-RLD remains unclear: a retrospective study of 90 patients with autoimmune pancreatitis reported pulmonary involvement in 51.2% (13), whereas another study found that biopsy-proven IgG4-RLD accounted for only approximately 0.2% of all pulmonary pathology specimens (14). This discrepancy likely reflects the invasiveness of lung biopsy and the fact that nonspecific findings may be misclassified without pathological confirmation. Intrathoracic lymphadenopathy is more common, detectable in half or more of IgG4-RD patients (13).

The clinical manifestations of IgG4-RLD are often nonspecific, and more than half of patients are asymptomatic at onset, with lesions detected incidentally on imaging (15, 16). Characteristic radiographic findings include nodules, masses, ground-glass opacities, alveolar-interstitial lesions, and bronchovascular bundle thickening (17). Among these, nodular lesions are particularly prevalent. A study of 89 patients with definite IgG4-RD found that 50 (56.2%) had radiologically confirmed pulmonary nodules, most of which were multiple (41/50) and bilateral (34/50) with regular margins (18). A recent single-center study of 20 IgG4-RLD patients reported that solid nodular patterns were the most frequent imaging finding, observed in 50% of cases (6). Importantly, these nodules can mimic malignancy—spiculated margins, lobulation, and pleural indentation have all been described in IgG4-RLD nodules (14)—making pathological confirmation essential.

Literature reports indicate that the symptoms and chest imaging findings of IgG4-RLD are non-specific, making it difficult to differentiate from pulmonary infections, tuberculosis, and malignant tumors, with a high misdiagnosis rate. In this case, given the patient’s elevated white blood cell count and CRP, chest CT showing plaque consolidation in the right middle and lower lobes, accompanying respiratory symptoms, and poorly controlled type 2 diabetes, we initially considered secondary infection due to impaired immunity. However, the patient showed no clinical improvement after antimicrobial therapy, and the pulmonary lesions progressively enlarged. The diagnosis was ultimately confirmed by pathological examination. A particularly noteworthy feature of this case is the normal serum IgG4 level. Most IgG4-RLD patients have elevated serum IgG4, with one study reporting elevated levels in 53 of 56 cases (94.6%) (19). However, serum IgG4 levels tend to be lower in patients with isolated pulmonary involvement compared with those with multi-organ disease (20). Importantly, it is well-recognized that a substantial proportion of patients with biopsy-proven IgG4-RD have normal serum IgG4 concentrations (21), and a normal level should not exclude the diagnosis. Moreover, elevated serum IgG4 has been reported in various other conditions, including cystic fibrosis (possibly associated with *Pseudomonas aeruginosa colonization*) (22), recurrent infections, autoimmune diseases, malignancies, and idiopathic interstitial pneumonia (23). Thus, reliance on serum IgG4 alone can be misleading.

Another distinctive aspect of this case is the presence of multiple mildly enlarged lymph nodes and elevated CRP, which prompted consideration of alternative diseases, such as multicentric Castleman disease (MCD). However, despite the presence of lymphadenopathy, a diagnosis of MCD was considered unlikely for several reasons. Clinically, the course of our case was distinctly different from that of typical MCD: CRP elevation was only mild (35.69 mg/L), fever was absent throughout the disease course, and the lymphadenopathy was mild and non-progressive; whereas typical MCD is characterized by a profound hyperinflammatory state with markedly elevated inflammatory markers (often CRP > 50–100 mg/L), persistent or progressive fever, night sweats, weight loss, and progressive lymphadenopathy. Furthermore, immunohistochemistry of the percutaneous lung biopsy specimen revealed an IgG4/IgG-positive cell ratio of approximately 42% and approximately 30 IgG4-positive plasma cells per high-power field; in contrast, the plasma cell variant of MCD typically manifests as polyclonal or monoclonal plasmacytosis, rather than the IgG4-dominant plasma cell infiltration observed in our case. Additionally, the patient showed an excellent response to corticosteroid monotherapy, with significant symptomatic improvement and near-complete resolution of the pulmonary lesions on follow-up imaging—a therapeutic response characteristic of IgG4-RD but not typical of MCD, which often requires IL-6-targeted therapies (e.g., siltuximab) for optimal disease control. We acknowledge that, given the mild and non-progressive nature of the lymphadenopathy, a lymph node biopsy was not performed, and in the absence of lymph node histopathology, a definitive tissue-based exclusion of MCD is not possible; nevertheless, multiple clinical features in our case strongly argue against a diagnosis of MCD. It is also worth noting that several studies have reported variably elevated CRP levels in patients with IgG4-RD (14, 24), further supporting that mild CRP elevation does not exclude the diagnosis.

Currently, no standardized diagnostic criteria specifically for IgG4-RLD have been established; the diagnosis of pulmonary involvement in IgG4-RD generally relies on the comprehensive diagnostic criteria for IgG4-RD. In clinical practice, the 2019 ACR/EULAR International Classification Criteria for IgG4-RD (25), based on a weighted scoring system, have demonstrated superior diagnostic performance compared with the traditional comprehensive diagnostic criteria. Although these criteria are primarily intended for clinical research, in the absence of established diagnostic criteria specifically for IgG4-RLD, and given their high specificity (98.0%) in the Chinese population (26), they provide a useful reference framework to support the diagnostic assessment in clinically suspected cases, particularly when combined with histopathological confirmation.

We applied the 2019 ACR/EULAR criteria for IgG4-RD to this case using the recommended three-step framework (25). First, the entry criterion was met, as the patient presented with characteristic involvement of typical organs (lung), and pathological examination of the lung biopsy confirmed an inflammatory process with lymphoplasmacytic infiltration. Second, no exclusion criteria were present: the patient had no clinical, serological, or pathological findings suggestive of other conditions that would preclude the diagnosis of IgG4-RD (e.g., no fever, no evidence of malignancy, no other disease-specific autoantibodies). Third, the weighted inclusion criteria were applied, yielding the following scores: bilateral submandibular gland enlargement (6 points), chest CT showing a focal lung mass, histopathological findings of abundant lymphocytic and plasma cell infiltration (4 points), immunohistochemical staining revealed a ratio of IgG4-positive to IgG-positive plasma cells of approximately 42% and approximately 30 IgG4-positive plasma cells per high-power field (14 points)—yielded a total score exceeding 20 points, fulfilling the classification criteria for IgG4-RD. In our case, the pulmonary involvement was attributed to IgG4-RD based on: (1) multi-organ involvement (lung and submandibular glands); (2) lung biopsy showing dense lymphoplasmacytic infiltration with an IgG4/IgG ratio of approximately 42% and approximately 30 IgG4-positive cells/HPF; (3) exclusion of infection, malignancy, and other interstitial lung diseases; and (4) an excellent response to corticosteroid therapy. Thus, despite several atypical features, the available evidence supports the classification of this case as IgG4-RLD.

IgG4-RLD generally responds favorably to corticosteroid therapy (8). Following diagnostic confirmation, the patient was started on prednisone 60 mg/day, based on her body weight (approximately 60 kg) and evidence of active disease—gradual enlargement of the lung lesion on follow-up CT. During follow-up, her symptoms improved dramatically, and imaging revealed near-complete resolution of the pulmonary lesions, confirming an excellent response to corticosteroid therapy. The patient was followed up through April 2026. Follow up chest CT demonstrated resolution of the pulmonary lesions with no evidence of recurrence, and she remained in good general condition. Although there is no consensus on the optimal corticosteroid regimen for IgG4-RD, most experts recommend an initial dose of 30–40 mg/day with a slow taper over several weeks, and combination with immunosuppressants may be considered for refractory or relapsing cases (8, 27).

In summary, IgG4-RLD is a rare disease with nonspecific manifestations, and insufficient awareness frequently leads to misdiagnosis. We acknowledge the limitations of this case, including the absence of lymph node biopsy and BAL fluid analysis for plasma cells and IgG4. Nevertheless, the diagnosis was established based on histopathological findings, characteristic imaging features, and a favorable response to corticosteroids. Although the disease generally responds well to corticosteroids, its long-term prognosis remains unclear. By sharing this case, we aim to raise awareness of IgG4-RLD in patients with unexplained pulmonary lesions and multi-system involvement, thereby reducing diagnostic delays. Further studies are needed to elucidate its etiology, risk factors, long-term complications, and optimal management.

## Data Availability

The original contributions presented in the study are included in the article/supplementary material. Further inquiries can be directed to the corresponding authors.

## References

[1] Peyronel F Della-Torre E Maritati F Urban M Bajema I Schleinitz N . IgG4-related disease and other fibro-inflammatory conditions. Nat Rev Rheumatol. (2025) 21:275–90. doi: 10.1038/s41584-025-01240-x 40195520

[2] Martínez-Valle F Orozco-Gálvez O Fernández-Codina A . Update in ethiopathogeny, diagnosis and treatment of the IgG4 related disease. Med Clin (Barc). (2018) 151:18–25. doi: 10.1016/j.medcle.2018.05.005 29241876

[3] Wallace Z Zhang Y Perugino C Naden R Choi H Stone J . Clinical phenotypes of IgG4-related disease: an analysis of two international cross-sectional cohorts. Ann Rheum Dis. (2019) 78:406–12. doi: 10.1136/annrheumdis-2018-214603 30612117 PMC6996288

[4] Hamano H Kawa S Horiuchi A Unno H Furuya N Akamatsu T . High serum IgG4 concentrations in patients with sclerosing pancreatitis. N Engl J Med. (2001) 344:732–8. doi: 10.1056/nejm200103083441005 11236777

[5] Duvic C Desrame J Lévêque C Nedelec G . Retroperitoneal fibrosis, sclerosing pancreatitis and bronchiolitis obliterans with organizing pneumonia. Nephrol Dial Transplant. (2004) 19:2397–9. doi: 10.1093/ndt/gfh050 15299101

[6] Han D Ying C Zhao T Tong Q Liu W . Clinical characteristics of IgG4-related lung disease: a single-center based experience. Clin Rheumatol. (2025) 44:4159–68. doi: 10.1007/s10067-025-07678-4 40926182

[7] Zen Y Nakanuma Y . IgG4-related disease: a cross-sectional study of 114 cases. Am J Surg Pathol. (2010) 34:1812–9. doi: 10.1097/PAS.0b013e3181f7266b 21107087

[8] Ryu J Sekiguchi H Yi E . Pulmonary manifestations of immunoglobulin G4-related sclerosing disease. Eur Respir J. (2012) 39:180–6. doi: 10.1183/09031936.00025211 21719489

[9] Dragos C Joseph C Elwell H Dey M Kouranloo K . Pulmonary manifestations, treatments and outcomes of IgG4-related disease-a systematic literature review. Rheumatol Int. (2024) 44:1875–86. doi: 10.1007/s00296-024-05611-7 38769126 PMC11393110

[10] Ogoshi T Kido T Yatera K Oda K Nishida C Yamasaki K . Incidence and outcome of lung involvement in IgG4-related autoimmune pancreatitis. Respirology. (2015) 20:1142–4. doi: 10.1111/resp.12599 26182953

[11] Umehara H Okazaki K Kawa S Takahashi H Goto H Matsui S . The 2020 revised comprehensive diagnostic (RCD) criteria for IgG4-RD. Mod Rheumatol. (2021) 31:529–33. doi: 10.1080/14397595.2020.1859710 33274670

[12] Raina A Yadav D Krasinskas A McGrath K Khalid A Sanders M . Evaluation and management of autoimmune pancreatitis: experience at a large US center. Am J Gastroenterol. (2009) 104:2295–306. doi: 10.1038/ajg.2009.325 19532132 PMC6583795

[13] Fujinaga Y Kadoya M Kawa S Hamano H Ueda K Momose M . Characteristic findings in images of extra-pancreatic lesions associated with autoimmune pancreatitis. Eur J Radiol. (2010) 76:228–38. doi: 10.1016/j.ejrad.2009.06.010 19581062

[14] Liu J Liu Y Shen X He Z Yu T Pang L . Clinicopathological characteristics of IgG4-related lung disease. BMC Pulm Med. (2021) 21:413. doi: 10.1186/s12890-021-01781-3 34911521 PMC8672518

[15] Inoue D Zen Y Abo H Gabata T Demachi H Kobayashi T . Immunoglobulin G4-related lung disease: CT findings with pathologic correlations. Radiology. (2009) 251:260–70. doi: 10.1148/radiol.2511080965 19221056

[16] Zen Y Inoue D Kitao A Onodera M Abo H Miyayama S . IgG4-related lung and pleural disease: a clinicopathologic study of 21 cases. Am J Surg Pathol. (2009) 33:1886–93. doi: 10.1097/pas.0b013e3181bd535b 19898222

[17] Muller R Ebbo M Habert P Daniel L Briantais A Chanez P . Thoracic manifestations of IgG4-related disease. Respirology. (2023) 28:120–31. doi: 10.1111/resp.14422 PMC10100266 36437514

[18] Xie Y Xiong A Marion T Liu Y . Lung nodules and IgG4 related disease: a single-center based experience. BMC Pulm Med. (2020) 20:218. doi: 10.1186/s12890-020-01250-3 32795329 PMC7427868

[19] Zhang W Zhang M Gong P Pan F Sun K Bao J . Clinical features of IgG4-related lung disease. Zhonghua Yi Xue Za Zhi. (2023) 103:1417–22. doi: 10.3760/cma.j.cn112137-20221025-02226 37150695

[20] Sun X Liu H Feng R Peng M Hou X Wang P . Biopsy-proven IgG4-related lung disease. BMC Pulm Med. (2016) 16:20. doi: 10.1186/s12890-016-0181-9 26809651 PMC4727342

[21] Carruthers M Khosroshahi A Augustin T Deshpande V Stone J . The diagnostic utility of serum IgG4 concentrations in IgG4-related disease. Ann Rheum Dis. (2015) 74:190–5. doi: 10.1136/annrheumdis-2013-204907 24651618

[22] Clerc A Reynaud Q Durupt S Chapuis-Cellier C Nové-Josserand R Durieu I . Elevated IgG4 serum levels in patients with cystic fibrosis. PloS One. (2017) 12:e0181888. doi: 10.1371/journal.pone.0181888 28922375 PMC5602527

[23] Ebbo M Grados A Bernit E Vély F Boucraut J Harlé J . Pathologies associated with serum igG4 elevation. Int J Rheumatol. (2012) 2012:602809. doi: 10.1155/2012/602809 22966232 PMC3433130

[24] Lv X Gao F Liu Q Zhang S Huang Z Zhu Y . Clinical and pathological characteristics of IgG4-related interstitial lung disease. Exp Ther Med. (2018) 15:1465–73. doi: 10.3892/etm.2017.5554 29434730 PMC5776625

[25] Wallace Z Naden R Chari S Choi H Della-Torre E Dicaire J . The 2019 American College of Rheumatology/European League Against Rheumatism classification criteria for IgG4-related disease. Arthritis Rheumatol. (2020) 72:77–87. doi: 10.1136/annrheumdis-2019-216561 31793250

[26] Liu Z Nie Y Peng Y Lu H Zhang P Li J . The external validation of the 2019 ACR/EULAR classification criteria for IgG4-related disease in a large cohort from China. Semin Arthritis Rheum. (2023) 61:152202. doi: 10.1016/j.semarthrit.2023.152202 37167774

[27] Khosroshahi A Wallace Z Crowe J Akamizu T Azumi A Carruthers M . Second international symposium on igG4-related disease. International consensus guidance statement on the management and treatment of igG4-related disease. Arthritis Rheumatol. (2015) 67:1688–99. doi: 10.1002/art.39132 25809420

